# Meta learning based residual network for industrial production quality prediction with limited data

**DOI:** 10.1038/s41598-024-62174-0

**Published:** 2024-05-25

**Authors:** Yiguan Shi, Yazhao Cao, Yong Chen, Longjie Zhang

**Affiliations:** 1 School of Mechanical Engineering, Beijing Institute of Technology, Beijing, 100081 China; 2 School of Automation Engineering, University of Electronic Science and Technology of China, Chengdu, 611731 China; 3 China South Industries Group Automation Research Institute Co. Ltd, Mianyang, 621000 China

**Keywords:** Limited data, Production quality prediction, Meta learning, Residual network, Effective channel attention, Engineering, Mathematics and computing

## Abstract

Due to the challenge of collecting a substantial amount of production-quality data in real-world industrial settings, the implementation of production quality prediction models based on deep learning is not effective. To achieve the goal of predicting production quality with limited data and address the issue of model degradation in the training process of deep learning networks, we propose Meta-Learning based on Residual Network (MLRN) models for production quality prediction with limited data. Firstly, the MLRN model is trained on a variety of learning tasks to acquire knowledge for predicting production quality. Furthermore, to obtain more features with limited data and avoid the issues of gradient disappearing or exploding in deep network training, the enhanced residual network with the effective channel attention (ECA) mechanism is chosen as the basic network structure of MLRN. Additionally, a multi-batch and multi-task data input approach is implemented to prevent overfitting. Finally, the availability of the MLRN model is demonstrated by comparing it with other models using both numerical and graphical datasets.

## Introduction

Production quality has been the enduring focus of industrial production processes, attracting numerous researchers seeking improvement strategies. Firstly, the Production Quality Prediction (PQP) model is a fundamental tool for enhancing production quality and has played a significant role in industrial production^1^. The function of PQP is to evaluate and judge the quality level of products based on all aspects of their impact. By identifying the main factors influencing production quality through PQP, improvement suggestions can be offered to the industrial production process^2^. In modern industrial manufacturing systems, data-driven intelligent PQP methods have been rapidly developed due to advancements in sensor techniques and the accumulation of industrial big data^3,4^. However, acquiring adequate product data in industrial production can be costly, and traditional deep learning models that rely on large amounts of data may not be suitable for certain industrial applications. Therefore, PQP with limited data has become a hot topic for research in modern industrial systems^5^.

Early, PQP was mainly based on traditional machine learning methods, such as partial least squares^6^, bayesian^7^ and decision tree^8^. These methods greatly reduce the workload compared to directly identifying product defects with the naked eye. However, PQP based on traditional machine learning is less accurate. In recent literature, deep learning-based intelligent fault diagnosis methods have led to a series of breakthroughs due to its attractive characteristic that directly learns the high-level and hierarchical representations from massive raw data^9^. But in practical industry, the data available for training is insufficient for deep learning model training. The scarcity of data often leads to overfitting problems and hinders the training of high-precision quality prediction models. How to solve production quality prediction with only a small amount of data^10^ and obtain satisfactory results needs to be paid attention to. Therefore, few-shot production quality prediction has become a critical issue to address in modern industrial systems.

In the literature, the solutions to few-shot learning include domain generalization^11^ and transfer learning^12^. After the application of these methods in other fields, including object detection^13^ and fault classification^14^, there have been some successful examples of few-shot learning methods^15^ in production quality prediction. However, in most cases, the above model can only be trained from scratch, and then fine-tuning is used to learn new tasks, which limits their adaptability in actual industrial production.

Meta-learning is a highly versatile learning approach tailored for few-shot problems, emphasizing the acquisition of learning adaptability rather than focusing solely on learning itself^16^. Meta-learning is aimed at the characteristics of the traditional neural network model, which has insufficient generalization performance and poor adaptability to new kinds of tasks. By harnessing learning adaptability, minimal adjustments suffice to adapt to new tasks encountered in practical industrial settings. Notably, meta-learning doesn't directly learn a predictive mathematical model but rather learns how to generalize learning of such models. Model-Agnostic Meta-Learning (MAML)^17^, as an optimization-centric meta-learning technique, aims at discovering an initial parameter set adaptable to new tasks. This empowers the model to refine its performance through gradient updates on small amounts of data from new tasks.

Based on these models, to achieve the purpose of PQP with limited data and alleviate the problem of model degradation in deep learning network training process, the meta-learning based on residual network (MLRN) approach is proposed in this paper. Below, we list our main contributions:

(i) To avoid overfitting in the limited data condition, an intelligent production quality prediction model MLRN is proposed. Compare with traditional machine learning, MLRN shows superiority in working out production quality analysis of limited data by finding the robust knowledge adaptability initialization parameters.
(ii) To extract more useful features from limited product quality data, an improved residual network structure was adopted in MLRN. By replacing the ReLU activation function with LeakyReLU, the issue of neuron death was addressed, thereby enhancing information propagation and improving the prediction accuracy of the MLRN model.
(iii) To further extract more effective features, this paper integrates an efficient channel attention mechanism into the improved residual network module, effectively enhancing MLRN's capability in extracting subtle features while reducing redundant information. This integration strengthens the generalization ability of the MLRN model.

The rest of this article is arranged as follows. In section “Literature review”, the few shot learning model based on meta-learning is introduced in detail. The problems associated with quality analysis of limited data of products are raised in Section “Problem description”. In Section “Proposed method”, the algorithm principle and network framework proposed MLRN method are introduced. In Section “Results analysis”, two experimental simulations are implemented to verify the effectiveness of the proposed MLRN. Finally, the conclusion of this paper is given in Section “Conclusion”.

## Literature review

Research literature on few-shot learning exhibits great diversity, spanning from data augmentation to supervised meta-learning. In this paper, we focus on the meta-learning-based methods most relevant to ours. In the field of machine learning, numerous meta-learning and meta-reinforcement learning algorithms have been proposed. This section will introduce the research status of meta-learning in detail from four aspects: weight-based meta-learning, metrics-based meta-learning, model-based meta-learning, and data enhancement-based meta-learning.

### Weight-based meta-learning

The weight-based meta-learning method enables the network to learn to initialize effective weights by itself, eliminating the need to manually configure the initial weight parameters of the model. The optimal performance can be obtained by learning the experience of historical tasks and training with a small number of new task samples. Ravi et al.^18^ proposed a meta-learner based on LSTM. The algorithm utilizes the LSTM meta-learning model to learn precise optimization algorithms and trains another neural network classifier in a small sample mechanism. After training, the model can provide a good set of optimization schemes. In addition, the form of the meta-learner model allows it to learn a task general initialization for the classifier. This common initialization is used to capture the basic knowledge shared across all tasks. The model-agnostic meta-learning (MAML) algorithm proposed by Finn et al*.*^17^ The key of MAML is to maximize the sensitivity of the loss function of the new task to the initial weight, independent of the type of model, and optimize the parameters in the direction of the gradient vector sum of each task to estimate the optimal parameter solution of the new task, so as to achieve the optimal performance along the gradient direction quickly. In addition, a self-adaptation graph attention network based on meta-learning is introduced by Long et al.^19^. The model can be quickly adapted to new tasks with limited data by meta learning algorithm, and it also has strong meta-knowledge learning ability because of the effective strategy. This method is suitable for many tasks such as regression, classification and reinforcement learning, but there are some problems such as quadratic gradient instability.

### Metrics based meta-learning

Metric-based meta-learning is an extension of metric learning within the field of meta-learning. Specifically, it primarily learns an embedding mapping function from inputs to features and then utilizes it to compute similarity metrics between tasks. An effective strategy with training an unbiased meta-learning algorithm was developed in^20^, which sorted out problems of target preference and few-shot under the meta-learning paradigm. Snell et al*.* introduced the prototype network^21^, which is based on the fundamental assumption that within a dataset, there exists a prototype point for each distinct category. Samples closer to this prototype point in the dataset are more likely to share the same label as the label corresponding to that prototype point. Reshkin et al*.*^22^ proposed Metric scaling to enhance the performance of few-shot classification algorithms. This method learns a distance scaling factor to ensure that the output metric falls within an appropriate range. Furthermore, to address the challenge of model degradation during the training process when adjusting hyperparameters, a task-sequencing meta-learning (TSML) approach for fault diagnosis with minimal occurrences is proposed in^23^. This method entails the development of a meta-learning strategy that involves task ordering and the exploration of suitable initialization parameters. In addition to these, there are several other metric-based meta-learning methods, such as extended versions of prototype networks^24^, multimodal approaches^25^, and so forth. In summary, metric-based methods offer flexibility, allowing for various choices from feature extractors, similarity metrics, loss functions, to hyperparameters. They also exhibit good average performance and are relatively straightforward to implement.

### Model-based meta-learning

Model-based meta-learning is a technique that uses additional model optimization algorithms to enhance the training process of neural networks**.** Memory-Augmented Neural Networks (MMANN), inspired by the Neural Turing Machine, undergo a series of adjustments in training setups and memory retrieval mechanisms, transitioning from location-based memory access to content-based access^26^. This methodology facilitates the gradual acquisition of abstract methods through gradient descent, enabling the extraction of meaningful representations from raw data. Moreover, MMANN enables swift binding of unfamiliar information post a single demonstration, thanks to an external memory module. In order to solve the high complexity of the training and fine-tuning stages, an embedded model is designed in^27^ as a transportable feature extractor, and then the support vector regression is fitted, which does not necessarily require labeled data in pre-training, and does not require fine tuning of the pre-training model in adaptive. In addition, Sun et al*.*^28^ introduced the meta-transfer learning (MTL) method, which utilizes deep neural networks pretrained on large-scale datasets to address few-shot learning challenges. This approach enhances learning efficiency by transferring learned weights and introducing a hard task meta-batch scheme, resulting in superior performance.

### Data enhancement-based meta-learning

As the most direct approach to meta-learning, data augmentation-based algorithms aim to address the challenges of insufficient sample quantity and low feature dimensionality in small-sample learning by employing various data augmentation techniques to increase the number of samples. Dixit et al.^29^ proposed a new model based on the combination of condition assisted classifier Generative Adversarial Network (GAN) framework and meta learning, which can generate high-quality synthetic data through GAN for the model. In^30^, a straightforward and versatile framework named MetaGAN is introduced. This framework improves upon traditional meta-learning methods by incorporating a task-based adversarial generator. The primary concept involves refining the model's decision boundary more effectively during the discrimination between real and fake data, thereby enhancing its feature extraction capabilities. Additionally, MetaGAN has the capability to extend supervised meta-learning methods and seamlessly handle unlabeled data. It is proposed that generating synthetic data can enhance the diversity of samples in^31^, thereby facilitating improved meta-learning. The paper argues that through generation structures, environmental factors such as posing and lighting conditions can be transferred to new samples, consequently producing new samples with diverse variations and effectively expanding the dataset.

## Problem description

**Figure 1:**
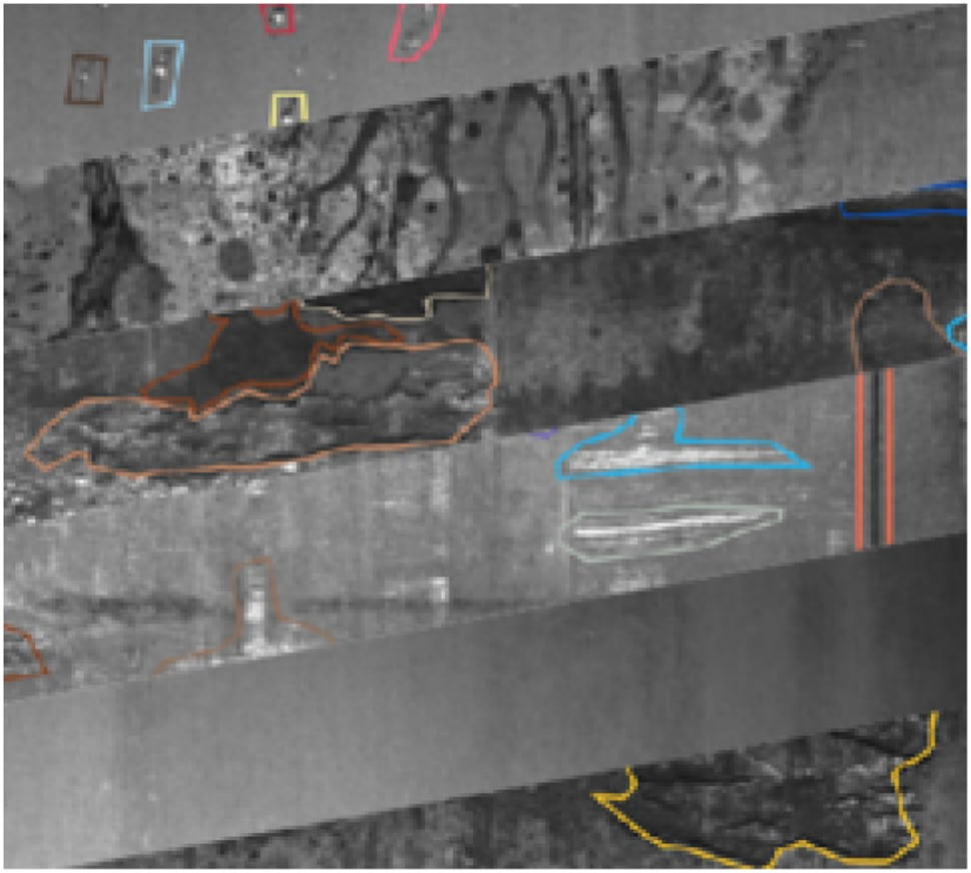
Surface defects of steel plates.

Current researches on PQP are mostly based on deep learning. Its principle is to establish PQP model by extracting features from the magnanimity raw product data directly. But the problem of less negative sample data always exists in industrial production. On the one hand, in the actual production process, the product runs normally most of the time with few failures. As a result, there are very few negative samples. On the other hand, the failure data collected is always not labeled, it requires a lot of labor costs to label. Therefore, it is difficult to acquire enough failure data to train the deep network^23^. Take PQP in steel plate as an example. There are 7 types of steel plates faults and 27 independent variables in Steel Plates Faults Data Set (SPF), (Dataset provided by Semeion, Research Center of Sciences of Communication, Via Sersale 117, 00,128, Rome, Italy) it is a numerical dataset. Fig. 1 shows the steel surface fault defects .The number of samples for each steel plates faults is shown in Table 1, where P stands for pasty, Z stands for “Z_Scratch fault”, K stands for “K_Scatch fault”, S stands for “Stains fault”, D stands for “Dirtiness fault”, B stands for “Bumps fault” and O stands for “Other fault”. Our goal is to train a model on SPF dataset, which can be used to predict production quality for new few-shot samples of steel plates.

**Table 1:** The number of samples for each steel plates faults.

style	P	Z	K	S	D	B	O
number	158	190	391	72	55	402	673

The steel plate fault data set is represented by D. We take the 27 independent variables as input $x$, and take their fault type as lael $y$. By analyzing the steel plates faults data set, we find that 27 independent variables have different dimensional, if the model is trained directly with raw data, the prediction accuracy of the model may not be high. Therefore, it is usually necessary for data preprocessing and normalization before the data analysis in the field of machine learning. Data preprocessing and normalization can not only enhance model prediction accuracy and convergence speed, but also improve model learning ability. A common method of data normalization is the Min–Max Normalization^24^, which convert the method of linearization of raw data to the range is shown by formula (1):

1 $$ x^{\prime } = \frac{{x - x_{\min } }}{{x_{\max } - x_{\min } }} $$

where $x$ represents the value of a single data, $x_{\min }$ is the minimum value of the column in which the data resides, and $x_{\max }$ is the maximum value of the column in which the data resides. After normalization of the data, the dataset of the steel plate is represented as $D = \{ (x_{1} ,y_{1} ), \ldots ,(x_{n} ,y_{n} )\}$. We need to optimize the parameter $\theta$ to predict a model $y = f_{\theta } (x)$ by formula (2):

2 $$ \theta^{*} = \arg \mathop {\min }\limits_{\theta } {\mathcal{L}}(D;\theta ,\omega ) $$

where ${\mathcal{L}}$ denotes loss function, $\omega$ denotes learning strategy, $\theta$ denotes product quality prediction model parameters. Because of the small amount of data in D and there is problem about gradient disappearance in deep network learning, traditional deep learning model cannot be used. The meta-learning based on residual network (MLRN) approach is adopted to solve PQP with limited data.

## Proposed method

### MLRN algorithm

In the current artificial intelligence algorithm, the training of the model is mainly based on data-driven. With a large amount of training data and gradient descent algorithm to optimize the model, the expected training task can be completed^32^. When artificial intelligence methods face a small amount of training data, model training will become difficult and prone to the problem of model over fitting. But for a kid, he can learn to recognize such new things with the limited data because of his learning ability. Meta-learning algorithm expects to design such a model that can learn new knowledge with a few training examples and it can apply previous learning experiences to new tasks. So meta-learning algorithm is also called to “learning to learn”^33^. MLRN insist the idea of meta learning, it acts on the new task by optimized parameters on the training task. The essence of MLRN algorithm is to train a model on a series of learning tasks, so that it can solve new small sample tasks through previous learning experience. Different from the traditional machine learning model training based on each piece of data, the training process of MLRN model is based on tasks, and the MLRN model learns knowledge between tasks through multi-batch and multi-task training. Therefore, the dataset $D$ is divided into $D_{train}$ and $D_{test}$ in MLRN and they all are divided into a series of tasks $D_{train} = \{ task_{1} , \cdots ,task_{n} \}$ and $D_{test} = \{ task_{1} , \cdots ,task_{m} \}$, where $task_{i} = \{ D_{train}^{i} ,D_{test}^{i} \}$. By training on these tasks, we need to predict a model:

3 $$ \mathop {\min }\limits_{\omega } = \mathop E\limits_{{{\rm T} \sim p(T)}} {\mathcal{L}}(D;\omega ) $$

where $p({\mathcal{T}})$ stands for the sum of tasks and $\omega$ stands for the learning strategy. We need to find a learning strategy $\omega$ that minimizes the value of the loss function for all tasks.

According to the above dataset, MLRN is divided into two layers: inner layer and outer layer, and there is a gradient updating algorithm in each layer. Among them, gradient update of inner layer refers to the gradient update of a single task on the temporary model, but does not affect the original model. The gradient update of the outer layer is the gradient update from one task to another, acting on the original model. To distinguish the data set of inner and outer layers, the training data and testing data of inner layer is called support set and query set, so each task is denoted as $task_{i} = \{ D_{{\sup {\text{port}}}}^{i} ,D_{{{\text{query}}}}^{i} \}$. And the training data and testing data of outer layer are still called training data and testing data. N-way K-shot is a common experimental setting in limited data scenario. N-way means that there are N categories in the training data of each inner task, and K-shot means that there are K labeled data under each category. MLRN algorithm look forward to obtain the initialization parameters by training $D_{train}$, and it can quickly fine tuning to achieve better result by training $D_{{t{\text{est}}}}$.

The above is expressed mathematically as follows: the goal of MLRN is to train a PQP model, which represented by a parameterized function $f_{\theta }$ with parameters $\theta$. The $\theta$ parameter is common to the inner and outer layers, and it is updated by two gradient updates. The inner layer calculates loss function by the support set and query set on the subtask, and then updated with the parameters $\theta$ to $\theta^{\prime}$ on the new task ${\mathcal{T}}_{i}$, which is the first gradient update:

4 $$ \theta_{i}^{\prime } { = }\theta - \alpha \nabla_{\theta } {\mathcal{L}}_{{{\mathcal{T}}_{i} }} (f_{\theta } ) $$

where $\alpha$ is the fixed hyperparameter of the inner layer and ${\mathcal{L}}_{{{\mathcal{T}}_{i} }} (f_{\varphi } )$ is the loss function of task ${\mathcal{T}}_{i}$. The outer layer calculates the loss function across tasks, and then updated with the parameters $\theta$ by stochastic gradient descent, which is the second gradient update,

5 $$ \theta \leftarrow \theta - \beta \nabla_{\theta } \sum\limits_{{{\mathcal{T}}_{i} \sim p({\mathcal{T}})}} {{\mathcal{L}}_{{{\mathcal{T}}_{i} }} (f_{{\theta_{i}^{\prime } }} )} $$

where $\beta$ is the fixed hyperparameter of the outer layer and $\sum\limits_{{{\mathcal{T}}_{i} \sim p({\mathcal{T}})}} {{\mathcal{L}}_{{{\mathcal{T}}_{i} }} (f_{{\theta_{i}^{\prime } }} )}$ is the sum of loss functions of the batch task ${\mathcal{P}}({\mathcal{T}})$. The optimal parameter initialization $\theta$ of MLRN model is obtained according to the two gradient updates alternating optimization of Eqs. (5) and (6). Therefore, the goal of outer layer optimization is to minimum the loss function of the multi-task $p({\mathcal{T}})$:

6 $$ \mathop {\min }\limits_{\theta } \sum\limits_{{{\mathcal{T}}_{i} \sim p({\mathcal{T}})}} {{\mathcal{L}}_{{{\mathcal{T}}_{i} }} (f_{{\theta_{i}^{\prime } }} )} = \sum\limits_{{{\mathcal{T}}_{i} \sim p({\mathcal{T}})}} {{\mathcal{L}}_{{{\mathcal{T}}_{i} }} (f_{{\theta - \alpha \nabla_{\theta } {\mathcal{L}}_{{{\mathcal{T}}_{i} }} (f_{\theta } )}} } ) $$

As the above analysis, the essence of production quality analysis is fault classification. Therefore, the cross-entropy^34^ function is chosen as the loss function, which is expressed by formula (7):

7 $$ {\mathcal{L}}_{{{\mathcal{T}}_{{\text{i}}} }} (f_{\varphi } ) = \sum\limits_{{x^{(j)} ,y^{(j)} \sim {\mathcal{T}}_{i} }} {y^{(j)} \log f_{\varphi } } (x^{(j)} ) + (1 - y^{(j)} )\log (1 - f_{\varphi } (x^{(j)} )) $$

### Enhanced residual connection

For few-shot learning, the lack of data will inevitably lead to the low prediction accuracy of the quality model. Therefore, the existing limited data should be used as much as possible to explore and excavate more features. Studies show that the depth of the network can help the model fit more complex sample distributions and improve the robustness of the model. When the number of network layers is increased, more complex feature patterns can be extracted from the network, because the training process of the model is the process of adjusting parameters, the deeper the layer number is, the more adjustable parameters are, which means the greater the degree of freedom of adjustment and the better fitting of the complex objective function. So theoretically better results can be obtained when the model is deeper. Consequently, we can enhance the prediction accuracy of the model by increasing the number of layers in the MLRN network structure. In neural network training, the deeper the network is, the more parameters the model needs to learn and the more data it needs to train. Otherwise, insufficient data in deep learning will lead to overfitting. However, with the help of multi-batch and multi-task training characteristics of meta-learning, the problem of overfitting caused by insufficient data in deep learning can be avoided, which is also the advantage of MLRN model.

In the traditional meta-learning model, convolutional neural network is used as the learning framework. To make the model have better prediction accuracy, it is necessary to increase the depth of the network architecture of the MLRN model. MLRN model training is based on back propagation, and the process of passing errors forward from the final layer is a form of continuous multiplication. Therefore, with the increase of the number of neural network layers, there may have some problems, such as gradient disappearance and gradient explosion. To solve the above problems, the residual network was selected as basic network in MLRN. In structure, the "bottleneck" in residual learning unit is designed to decrease the amount of model parameters and increase network depth, so that the model has better feature learning ability and reduce the cost of calculation.

**Figure 2:**
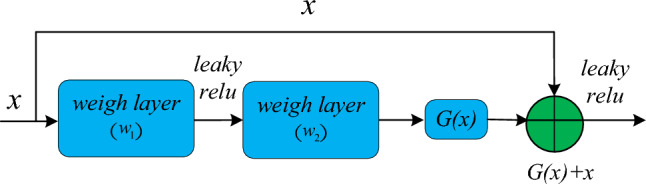
residual learning unit.

The basic idea of residual network is to introduce the concept of short-circuit connection, which makes it easier to optimize and short^35^. Several short-circuit connections are stacked together to form a residual learning unit. As the focus of this paper is on predicting product quality in small-sample data, the original network is based on ResNet18, utilizing conventional residual connection within the network. However, the original residual network exhibits excessive non-linear functions, such as ReLU activation functions, in the main pathway. This may hinder information propagation and impede the effective identification of product quality features. Therefore, to optimize information propagation efficiency in the network, LeakyReLU^36^ activation functions are used instead of the original ReLU within residual network connections, thereby enhancing the utilization of high-quality product features. LeakyReLU addresses the issue of neuron death while retaining all the advantages of ReLU activation functions. It allows for a small, non-zero gradient for negative inputs, ensuring the activation of neurons throughout the entire network. The improved residual learning unit is shown in Fig. 2.

Let's say the input to the model is $x$, the potential mapping achieved by using residual learning units is $G(x)$. Defining $G(x) = h(x) - x$ as the residual mapping, we have $h(x) = G(x) + x$, such that the residual unit approaches $G(x)$ infinitely, effectively allowing multiple nonlinear layers within the residual unit to approximate $h(x)$. Utilizing multiple nonlinear layers to achieve the residual mapping makes it easier for $h(x)$ to tend towards 0 compared to approximating the identity mapping using multiple nonlinear layers. Thus, the mathematical definition of a residual learning unit is:

8 $$ y = LR[x + G(x)] $$

where, $y$ is the output of the residual learning unit; $x$ is the input to the residual learning unit; $LR( \cdot )$ is the LeakyRelu activation function; $G(x)$ stands for the original mapping, and the formula of $G(x)$ is as follows:

9 $$ G\left( x \right) = w_{2} \times (LR(w_{1} \times x)) $$

where, $w_{1}$ and $w_{2}$ stand for the weight layer in the residual connection. When the accuracy of the model reaches saturation, the subsequent training of the model will limit the mapping of $G(x)$, and only an identity mapping between the output $y$ and the input $x$. Therefore, MLRN model based on residual network can increase the network depth without increasing the error, and improve the accuracy of model prediction.

### MLRN network structure based integrating ECA and enhanced residual connections

In few-shot learning, the information content of data is typically limited, making effective feature extraction crucial. Introducing attention mechanisms enables adaptive weight allocation within the network, allowing it to focus more on the most representative features within the samples. This enhances the efficiency and accuracy of feature extraction. This mechanism helps the network capture key features more effectively, reducing dependence on noisy data and thereby mitigating the risk of overfitting. This paper integrates efficient channel attention mechanism (ECA)^37^ with an improved residual network as the network architecture for product quality prediction models using small-sample data. The goal is to enhance model performance and interpretability.

**Figure 3:**
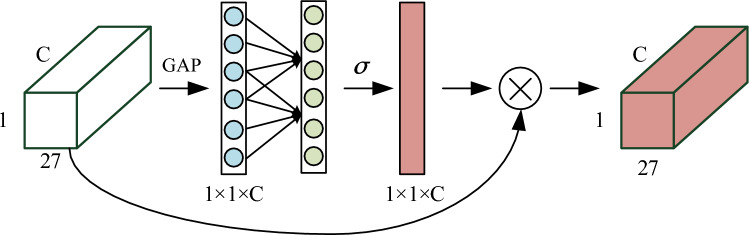
Efficient channel attention mechanism structure.

The structure of the ECA, as shown in Fig. 3, is designed to handle features of size 27 × 1 × C (where C represents the number of input channels). Upon receiving such features, the module initially employs a global average pooling layer to aggregate features without altering their dimensions. Subsequently, a one-dimensional convolutional layer is utilized for learning, allowing for weight sharing. This convolutional layer incorporates a hyperparameter, denoted as $k$, representing the kernel size, which signifies the coverage rate of local cross-channel interactions. This coverage rate is adaptively determined based on the mapping of the channel dimension C. Next, the learned weights undergo redistribution through the sigmoid activation function. Finally, the resulting 1 × 1 × C feature is aggregated with the original feature to obtain a new attentional feature, thereby significantly enhancing the model's ability to learn attention.

The local cross-channel interaction strategy without dimensionality reduction can complete the information exchange by nonlinear mapping adaptive one-dimensional convolution. As shown in Eq. (10).

10 $$ w = \sigma (C_{k} (x)) $$

where, $w$ is the weight; $\sigma$ stands for nonlinear mapping; $C_{k}$ represents one-dimensional convolution of $k$ parameter information; $x$ stands for the input of data. The weights of one-dimensional convolution are interleaved, that is, cross-channel, and exist in a group of groups. The number of weights in a group depends on the size of the convolution kernel $k$. $k$ is determined adaptively by formula (11).

11 $$ k = \psi (C) = \left| {\frac{lbc}{\gamma } + \frac{b}{\gamma }} \right|_{odd} $$

where, $\psi (C)$ represents the linear mapping relationship between the number of channels $C$; $k$ is the kernel size, representing the cross-channel interaction area; $C$ is the number of channels; $\left| \cdot \right|_{odd}$ stands for the nearest neighbor odd number; $\gamma$ represents the slope of the linear map, which is 2; $b$ is the intercept of the linear mapping and its value is 1; $lbc$ stands for the size of the data block.

**Figure 4:**
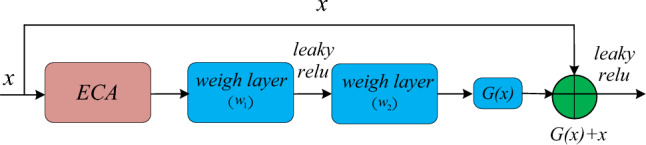
ECA + ResNet learning unit.

In order to balance the performance and complexity of MLRN algorithm, an effective channel attention mechanism is added to the improved residual network, which is named ECA + ResNet. The structure of ECA + ResNet is shown in the Fig. 4.

**Figure 5:**
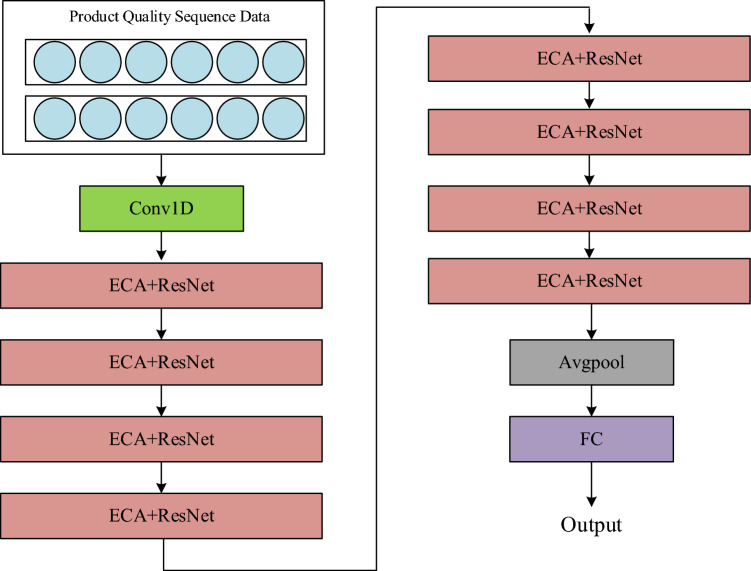
The basic network architecture of MLRN model.

**Figure 6:**
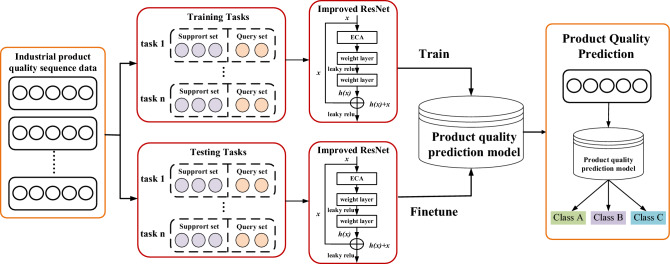
Working process of the MLRN for limited data intelligent Production quality Prediction on industrial production.

In this paper, the improved 18 layers residual network is chosen as the network architecture of MLRN model. It consists of one convolution layer, eight residual blocks (each residual block has two convolution layers and an efficient channel attention mechanism structure) and one full connection layer, which is named IEResNet18. Figure 5 shows the overall model framework. Figure 6 shows that working process of the MLRN for limited data intelligent production quality prediction on industrial production.

In addition, as shown in Algorithm 1, the learning process of MLRN model is as follows:

1. First, the Min-Max Normalization method is used to normalize the raw data and the 18 layers improved residual network IEResNet18 is constructed as the basic framework for MLRN.
2. Data set D is divided into training set $D_{train}$ and testing set $D_{{t{\text{est}}}}$.
3. Tasks in $D_{train}$ and $D_{{t{\text{est}}}}$ are sampled. We randomly pick N classes of all the categories and Q samples of each category, in which K samples are the training set in the inner layer, which is also called the support set, and the remaining Q-K samples are the testing set in the inner layer, which is also called the query set.
4. The cross-entropy loss function of Eq. (7) is selected as the loss function of each classification task, and according to the first gradient descent algorithm of Eq. (5), parameters of the task in the inner loop are optimized.
5. After the internal batch task parameters are updated in step 4), the outer layer parameters are updated according to the second gradient descent algorithm of Equation (6).
6. Repeat steps 4) and 5) to get the optimal parameter initialization $\theta$ of the MLRN model.

**Algorithm 1:**
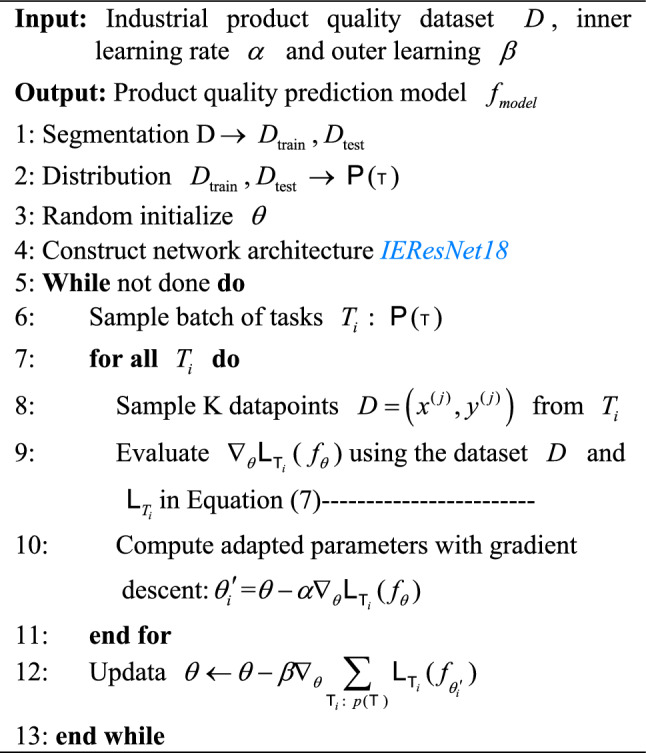
MLRN.

## Results analysis

In this part, to validate the MLRN model’s performance of production quality prediction on limited data, we did a series of experimental verification on the steel plates faults dataset. The Steel Plates Faults Data Set is a numerical data and there are 7 types of steel plates faults and 27 independent variables. Our simulation experiment process is as follows, which includes three parts: experimental parameter setup, experimental results and validation of model universality.

### Experimental parameter setup

N-way K-shot is a common experimental setup for the limited data scenario, so there are N*K training samples in each task. For the SPF dataset, we respectively performed 5-shot and 6-shot in the 5-way and 6-way scenarios to verify that MLRN predict accuracy in different sample sizes. Similarly, we randomly select 5 and 6 samples from each category as query sets. According to the above settings, the number of training epochs is 100 and the entire experimental process samples 10,000 training tasks and 100 testing tasks. As discussed in Section III, the learning process of MLRN algorithm is divided into two layers, the fixed hyperparameter α of the inner loop is 0.04 and the fixed hyperparameter β of the outer loop is 0.001. The training process inner update steps are set as 5, and the testing process steps for finetuning are set as 15.

As discussed in Section IV, improved residual network IEResNet18 is chosen as basic network architecture of MLRN to solve the degradation problem of deep neural network model. In the processing of training, all tasks are learned using the same basic network 18 layers improved residual network, which has one convolution layer, six enhanced residual block and one full connection layer. Then the following part consists of LeakyReLu nonlinearity and batch normalization. Because the essence of PQP is the fault classification problem, the cross-entropy function with multiple classification function is selected as the loss function, which is used to verify the quality of the model.

### Experimental result and analysis

In practical PQP, there have been other methods for the few-shot scenario analysis. To show the superiority of MLRN algorithm with limited data, two advanced methods, TSML^23^ and MTL^28^, are selected for contrast experiments, which the experimental parameters of two models are consistent with MLRN. The experimental parameters of the two compared advanced methods small-sample learning models are the same as those of MLRN, and all the tasks of the comparison algorithm are learned using the same network layer number, learning rate, loss function, training and testing steps.

**Figure 7:**
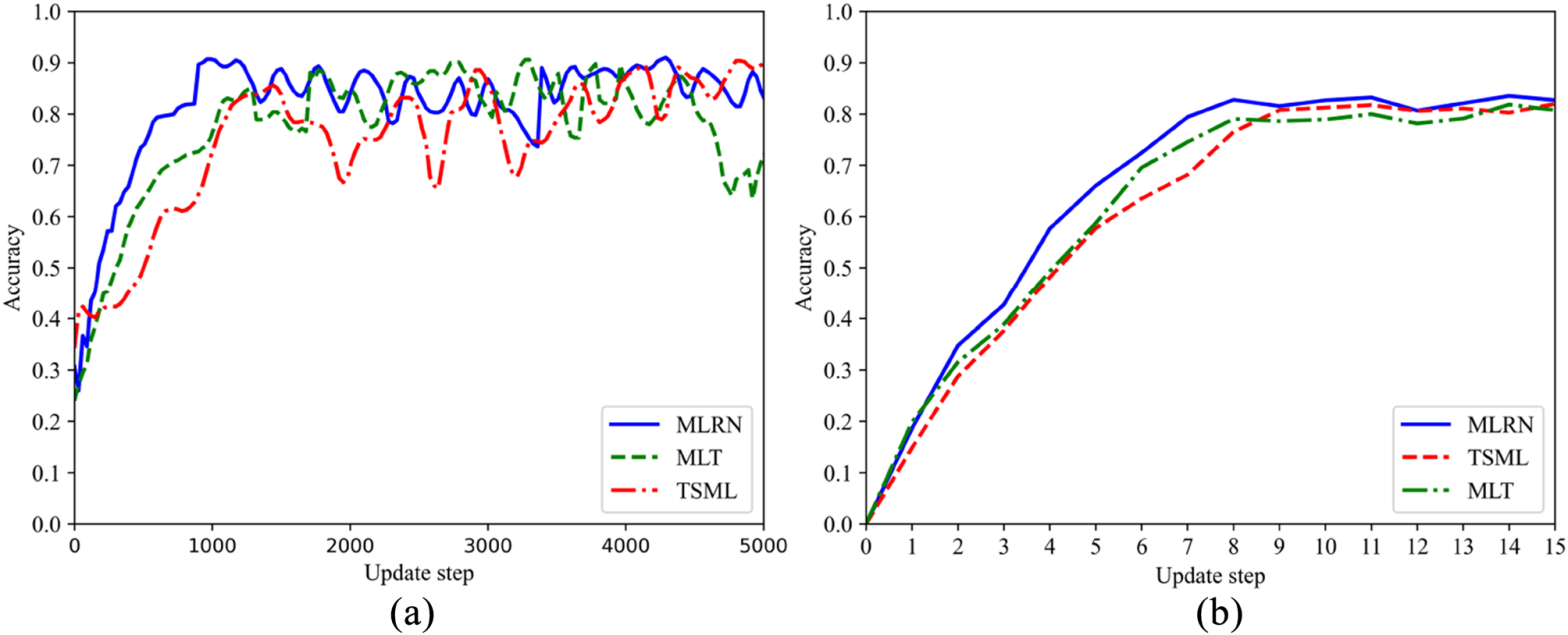
Accuracy of MLRN under 5-way 5-shot experimental conditions on the SPF dataset. (**a**) Meta-training stage; (**b**) Meta-testing stage.

Table 2 illustrates the predictive accuracy of the production quality models derived from three distinct algorithms trained on the SPF dataset. The accuracy of MLRN under different tasks are 82.69%, 83.58%, 78.33%, 80.37%, which is significantly higher than other models. MLRN outperforms the transfer meta-learning model MTL due to the fact that transfer learning involves learning the initial parameters from the source domain and fine-tuning them for tasks in the target domain. Thus, transfer learning focuses solely on the performance of the current task. In contrast, MLRN not only updates parameters but also seeks knowledge between tasks simultaneously, enabling it to consider all tasks in $D_{train}$ and $D_{test}$. Additionally, MLRN surpasses TSML models due to the enhanced residual network structure, which not only extracts more feature information from limited data but also improves fine-grained feature extraction performance through the ECA mechanism. Accuracy, although commonly used, cannot meet the requirements of all classification tasks. For example, there are 100 samples, of which 98 are positive samples and only 2 are negative samples. If the predictions are all positive after the input and output of the model, the accuracy of production quality prediction of the model is 98%, which is obviously not up to the prediction requirements, because the negative samples in the actual industrial production deserve more attention. Therefore, in addition to accuracy, precision and F1 score are also introduced for a more comprehensive evaluation. In these two indicators, MLRN is also superior to the baseline method, which shows that MLRN model also pays high attention to negative samples. To further explain the training process of MLRN, Fig. 7a illustrates the variation of accuracy with increasing update steps during the meta-training stage for both MLRN and the baseline algorithm. Similarly, Fig. 7b demonstrates the variation of accuracy with increasing update steps during the meta-testing stage for both MLRN and the baseline algorithm. It can be seen that the accuracy of the proposed MLRN method is improved more obviously and quickly.

**Table 2:** Experimental results of MLRN and the comparison algorithms on the SPF dataset.

Evaluation Metrics	Methods	5way		6way	
		5shot	6shot	5shot	6shot
Accuracy	MLT	0.7893	0.8011	0.7682	0.7724
	TSML	0.8126	0.8221	0.7621	0.7734
	MLRN	**0.8269**	**0.8358**	**0.7733**	**0.8037**
F1_score	MLT	0.7816	0.8042	0.7494	0.7512
	TSML	0.7955	0.8031	0.7501	0.7676
	MLRN	**0.8017**	**0.8094**	**0.7488**	**0.7711**
Precision	MLT	0.8159	0.8256	0.7568	0.7698
	TSML	0.8239	0.8314	0.7655	0.7866
	MLRN	**0.8357**	**0.8426**	**0.7854**	**0.8154**

Significant values are in bold.

### Ablation experiment result and analysis

Ablation experiment is a significant technique in deep learning for debugging and optimization, aiming to delve into the internal operations of models and understand the impact of different components on model performance by removing or replacing key components. In this section, we conduct comparative ablation experiments between the MLRN algorithm and VGG-based MAML (MAML-VGG) as well as ResNet-based MAML (MAML-ResNet) algorithms, to further validate the superiority of the improved residual network structure in the MAML model. For a fair comparison, the experimental parameters setting of two advanced models are the same as the MLRN, all tasks are learned by using the same network layers, learning rate, loss function, training and testing steps.

**Figure 8:**
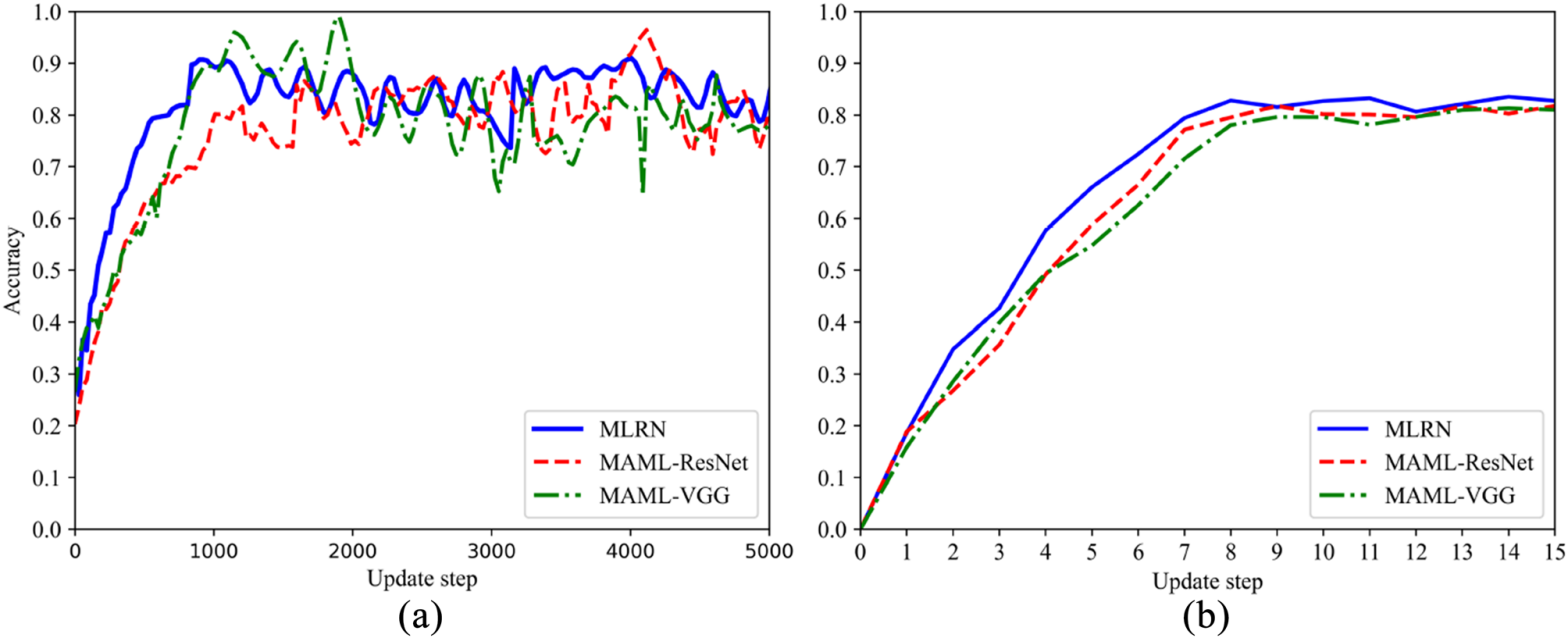
Accuracy of ablation experiment under 5-way 5-shot experimental conditions on the SPF dataset. (**a**) Meta-training stage; (**b**) Meta-testing stage.

 Table 3 shows the ablation results of MLRN and the comparison algorithm on the SPF dataset. Figure 8 shows the evolution of accuracy with increasing update steps during the training process for both MLRN and the ablation comparison algorithms. From Table 4 and Fig. 8, it is evident that the MLRN algorithm outperforms the ablation algorithms in terms of convergence speed and accuracy. This can be attributed to the improved residual network, which features inter-layer connections and shortcut connections, effectively mitigating the vanishing gradient problem and promoting information flow. Simultaneously, the ECA mechanism can adaptively adjust the importance of each channel in the features, enabling the network to focus more on extracting crucial features, thereby enhancing feature extraction capabilities and subsequently improving the performance of the MLRN model.

**Table 3:** Experimental results of MLRN and comparison algorithms on SPF dataset.

Evaluation Metrics	Methods	5way		6way	
		5shot	6shot	5shot	6shot
Accuracy	MLT	0.8016	0.8192	0.7683	0.7897
	TSML	0.7959	0.8081	0.7594	0.7823
	MLRN	**0.8269**	**0.8358**	**0.7733**	**0.8037**
F1_score	MLT	0.7898	0.7899	0.7394	0.7652
	TSML	0.7739	0.7860	0.7219	0.7568
	MLRN	**0.8017**	**0.8094**	**0.7488**	**0.7711**
Precision	MLT	0.8204	0.8244	0.7695	0.7921
	TSML	0.8176	0.8205	0.7605	0.7899
	MLRN	**0.8357**	**0.8426**	**0.7854**	**0.8154**

Significant values are in bold.

**Table 4:** Experimental results of MLRN and comparison algorithms on SPF dataset.

Evaluation Metrics	Methods	5way		6way	
		5shot	6shot	5shot	6shot
Accuracy	MLT	0.7313	0.7777	0.7294	0.7423
	TSML	0.7510	0.7717	0.7367	0.7577
	MLRN	**0.7884**	**0.8175**	**0.7504**	**0.7786**
F1_score	MLT	0.7481	0.7802	0.7107	0.7411
	TSML	0.7612	0.7738	0.7223	0.7519
	MLRN	**0.7783**	**0.8006**	**0.7344**	**0.7713**
Precision	MLT	0.7364	0.7818	0.7354	0.7451
	TSML	0.7563	0.7905	0.7472	0.7324
	MLRN	**0.7824**	**0.8017**	**0.7299**	**0.7517**

Significant values are in bold.

### Generalization of MLRN

The Steel Plates Faults Data Set is a numerical data. To verify the universality of MLRN model, the KTH-TIPS image database (KTS) (M. Fritz, E. Hayman, B. Caputo, and J.-O. Eklundh. The KTH-TIPS database. Available at www.nada.kth.se/cvap/databases/kth-tips) is used for training. The TIPS stand for textures under varying illumination, Pose and Scale. The KTS is a graphical data, it currently contains of 10 of those materials images. The database includes 810 grayscale images: 81 samples each of ten different kinds of typical surface defects.

**Figure 9:**
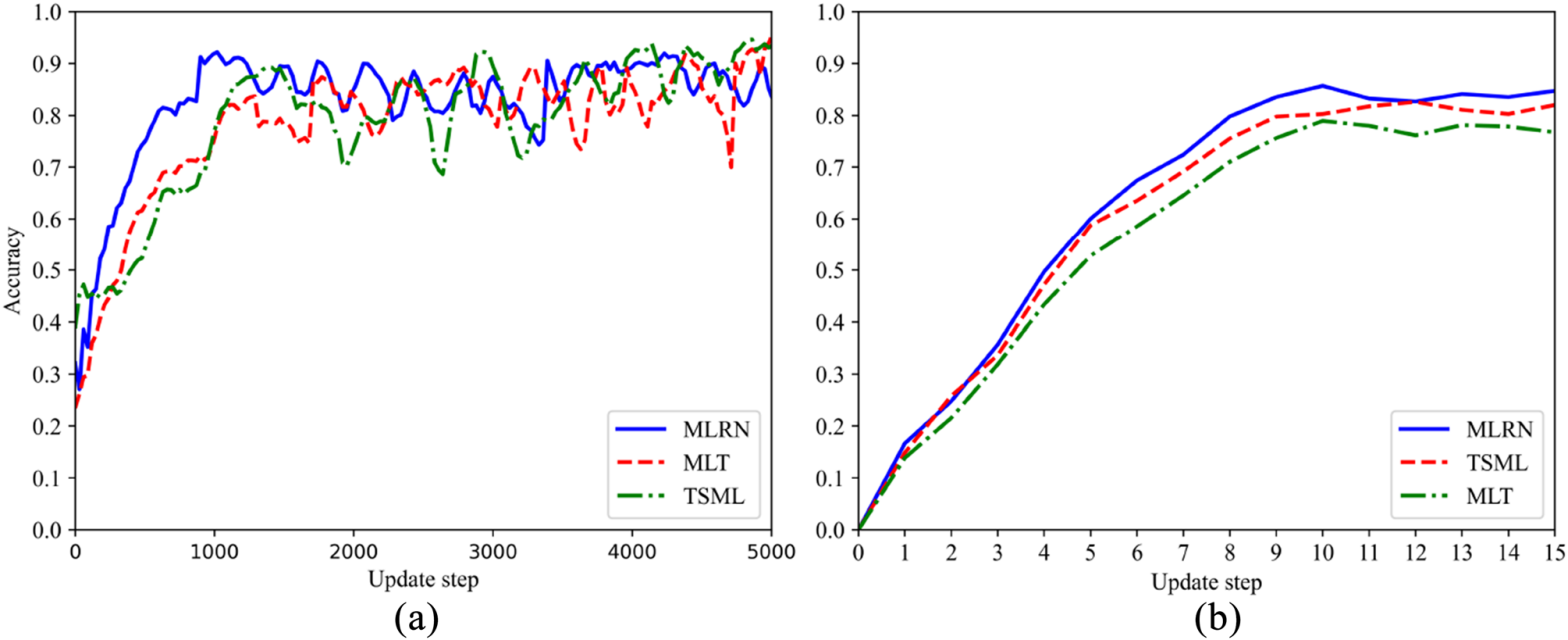
Accuracy of MLRN under 5-way 6-shot experimental conditions on KTS dataset. (**a**) Meta-training stage; (**b**) Meta-testing stage.

The parameters of experiment are consistent with the experimental (2). The accuracy curves during training process are shown in Fig. 9. By comparing the predicted accuracy curves of MLRN, TSML, and MLT, it is evident that MLRN outperforms the comparison algorithms in terms of prediction accuracy. This demonstrates that MLRN also exhibits excellent performance on graphical data. In order to verify that MLRN model also has better knowledge adaptability on new samples of the graphical dataset, Table 4 shows that the experimental results of the limited data on KTS. The accuracy of MLRN in 5 way are 78.84%, 81.75%, and the accuracy of MLRN in 6 way are 75.04%, 77.86%, which are also significantly higher than the other methods. Similarly, precision and *F*1 score are also introduced for a more comprehensive evaluation.

The above experimental results not only show that MLRN model is effective for graphical data, but also verify the universality of MLRN. Comparing the results of experiments on SPF and KTS datasets, although the prediction accuracy of the graphical data applied to MLRN is lower, the improvement is more significantly compared with the other two models, which proved that MLRN performs better on the KTS rather than that on the SPF. One of reason for this situation may be that there are more effective features of the graphical data, and the advantage of residual network is that can more features can be explored and mined, so the better classification effect and higher validity can be selected. And another reason may be that KTS has more categories, and the model can obtain more effective information to update the weight model in the training process. So MLRN in KTS can learn more knowledge between tasks.

## Conclusion

To address the challenge of limited data scenarios in industrial production and model degradation during deep network training processes, this paper investigates a meta-learning approach based on residual networks, referred to as MLRN, for predicting production quality with limited data. It is a model based on a meta-learning algorithm and a residual network framework. To demonstrate the effectiveness of MLRN in production quality compared to two other state-of-the-art models, MLRN exhibits superiority in convergence speed and prediction accuracy in PQP with limited data. The experimental results show that the MLRN model is effective in predicting products with limited data in industrial production. It could adapt to new categories using a small number of gradient update steps and has greater knowledge generalization ability. More importantly, the MLRN, based on the enhanced residual connection network structure with efficient channel attention mechanism, can extract more subtle features from limited data, alleviating the issue of model degradation, thus enhancing prediction accuracy.

Although the accuracy of MLRN model in production quality prediction is improved compared with other models, it can be seen from the above experimental results that MLRN model spends more time and resources in the training process. There are still many works that can be developed and improved upon in future study. On the one hand, we can combine the transfer learning network with meta learning algorithm, and join the generation confrontation network at the same time, it can generate more high-quality data for model training. On the other hand, the structure of residual network can be improved to reduce resource losses while maintaining model performance.

## Data Availability

The datasets generated and analysed during the current study are available in the MLRN repository, [https://www.kaggle.com/datasets/caotongx/mlrn-dataset].
